# TFIIB-Related Factor 2 Is Associated with Poor Prognosis of Nonsmall Cell Lung Cancer Patients through Promoting Tumor Epithelial-Mesenchymal Transition

**DOI:** 10.1155/2014/530786

**Published:** 2014-03-17

**Authors:** Yu Tian, Ming Lu, Weiming Yue, Lin Li, Shuhai Li, Cun Gao, Libo Si, Lei Qi, Wensi Hu, Hui Tian

**Affiliations:** Department of Thoracic Surgery, Qilu Hospital, School of Medicine, Shandong University, Jinan, Shandong 250012, China

## Abstract

In this study, we found that increased BRF2 protein expression was prevalent in NSCLC. Overexpression of BRF2 correlated with abnormal expression of E-cadherin, N-cadherin, and snail. Additionally, expression of BRF2 was found to be an independent prognostic factor in NSCLC patients. Furthermore, we showed that targeted knockdown of BRF2 expression could inhibit the migratory and invasive abilities of NSCLC cells and induced loss of the epithelial-mesenchymal transition of NSCLC cells. These results suggested that BRF2 overexpression in tumor tissues is significantly associated with the poor prognosis of NSCLC patients through promoting epithelial-mesenchymal transition (EMT) program.

## 1. Introduction

Lung cancer has remained the leading cause of cancer-related death worldwide for several years. Every year, there are 1.35 million new lung cancer cases in the world [1]. Nonsmall cell lung cancer (NSCLC) accounts for approximately 75–80% of cases of lung cancer [2, 3]. Despite the advances in early detection, radical cure operation, and multimodal therapeutic modalities in the past decades, the overall 5-year survival rate of lung cancer is only 15% [4]; recurrence and the emergence of metastases are major causes of therapeutic failure in cancer patients. Recent studies have shown that epithelial-to-mesenchymal transition (EMT) is associated with the acquisition of the malignant characteristics in NSCLC cells [5–9].

Epithelial-mesenchymal transition (EMT) initially occurs during normal embryonic development [10]; however, more recent reports suggest that epithelial-mesenchymal transition (EMT) is considered to be one of the major molecular mechanisms inducing tumor invasion and metastasis [11–13]. One of the hallmarks of EMT is the suppression of E-cadherin, a transmembrane protein essential for cell-cell adhesions, which is usually concomitant with the increase of mesenchymal N-cadherin expression, a mesenchymal-specific protein. Factors of snail zinc finger, Zeb, and bHLH families are known to suppress E-cadherin, which inhibit E-cadherin expression by binding to the proximal E-box of the E-cadherin promoter [14], thereby promoting the EMT process and tumor metastasis [15–17]. And overexpression of snail in epithelial cells has been shown to induce EMT and enhance invasion capacity [18, 19].

RNA polymerase (pol) III is responsible for the transcription of small, less than 300 nucleotides, untranslated RNAs including microRNAs (miRNAs) [20, 21]. Accurate transcription by RNA pol III requires TFIIIB, while BRF2 (TFIIB-related factor 2) is a component of TFIIIB. The regulation of pol III is integral to the growth control functions of RB, P53, and c-Myc, and TFIIIB activity is strictly regulated by Maf1, chemopreventive agents, oncogenes, and tumor suppressors [22–27]. Relationship between BRF2 gene and TFIIIB determines its important role in tumorigenesis and development process. BRF2 has been shown to be highly overexpressed in a variety of cancers including gastric, kidney, and melanoma cancers [28], and our previous studies have pointed out that BRF2 protein overexpression is common in early-stage ESCC and significantly correlated with tumor prognosis and relapse [29]. Recently, Lockwood et al. reported that overexpression of BRF2 could drive the expression of RNA pol III transcripts, contributing to squamous cell carcinoma tumorigenesis, and BRF2 has been identified as a novel lineage-specific oncogene in lung squamous cell carcinoma [30]. These studies suggest that BRF2 plays a complex role in lung cancer. Therefore, it is essential to further investigate the functional role of BRF2 in lung cancer invasion and metastasis.

Here, we evaluated the prognostic value of BRF2 expression in patients with resectable NSCLC and found that high expression of BRF2 in NSCLC predicted decreased overall 5-year survival and a higher risk of recurrence. Furthermore, disruption of BRF2 transcripts through small interfering RNA in NSCLC cells results in a reduced capacity of migration and invasion in vitro, inhibiting EMT related invasion in association with increased E-cadherin expression. We suggest that BRF2 may play an important role in the migration and invasion of NSCLC.

## 2. Materials and Methods

### 2.1. Patients and Tissue Specimens

A total of 77 consecutive patients who were diagnosed with NSCLC and treated with pulmonary lobectomy plus regional lymph node dissection from January 2005 through December 2006 at the Department of Thoracic Surgery, Qilu Hospital, were studied retrospectively. And they all had the clear pathological diagnosis without preoperative radiotherapy and chemotherapy. 37 cases of adjacent tissue samples were taken from about 0.5 cm away from the outer edge of the lung tumor tissues, and the other 43 cases were taken more than 5 cm from the tumor margin of normal lung tissues as a negative control. For RT-PCR and western blotting analysis, 14 matched pairs of tumors tissue and adjacent noncancerous tissue samples were obtained from pulmonary lobectomy specimens of patients diagnosed with NSCLC immediately after surgery between October 2010 and September 2011 in our department and stored at −80°C.

The clinical characteristics of the patients are summarized in Table 1. For all patients, histological type and grade of cancer cell differentiation were reevaluated and determined by the classification system of the World Health Organization modified in 2004, and postsurgical pathological staging was determined based on the international staging system. Clinical follow-up data was available for a minimum of 5 years or until death. Informed consent was obtained from all patients and this study was approved by the Ethics Committee of Qilu Hospital (Shandong, China).

### 2.2. Cell Lines and Culture Conditions

The human lung cancer cell lines A549 and SK-MES-1 were routinely obtained from the Shanghai Institute of Biochemistry and Cell Biology of China. The adenocarcinoma cell line A549 was cultured in Roswell Park Memorial Institute (RPMI) 1640 (Sigma, St Louis, USA) containing 10% FBS and the SK-MES-1 squamous cell carcinoma cell line was normally maintained in MEM supplemented with 20% fetal bovine serum. The cell lines were all cultured in 1% penicillin-streptomycin at 37°C in a 5% CO_2_ humidified cell culture incubator.

### 2.3. Immunohistochemistry

All specimens were collected during the surgery, fixed by 10% formalin, and embedded in paraffin. The tissues were cut as 4 *μ*m serial sections and then deparaffinized using xylene and rehydrated through an ethanol series to water. High-temperature antigen retrieval was carried out in citrate buffer for 25 min in a microwave oven. Then, the endogenous peroxidase enzyme activity was blocked using 3% H_2_O_2_ in methanol for 20 min at room temperature. The slides were then incubated with primary rabbit anti-BRF2 polyclonal antibody (1 : 400, Abcam, Cambridge, MA, USA), rabbit anti-E-Cadherin (1 : 200, Cell Signaling, Danvers, MA, USA), anti-N-Cadherin (1 : 200, Cell Signaling, Danvers, MA, USA), and anti-snail antibodies (1 : 100, Cell Signaling, Danvers, MA, USA) overnight at 4°C in a high humidity chamber, followed by incubation for 30 min at 37°C with biotinylated secondary antibodies and streptavidin-peroxidase complex. Finally, a 3,30-diaminobenzidine solution was added, and the slides were counterstained with hematoxylin and mounted with neutral balsam. For negative controls, sections were incubated with PBS instead of the primary antibodies.

### 2.4. Evaluation of Immunohistochemical Staining

All sections were reviewed independently by three independent observers blinded to all clinical and pathologic information. Discordant cases were resolved by choosing the value that is consistent between two observers or the average of the scores. A reproducible semiquantitative method that considered both staining intensity (0, negative; 1, weak; 2, moderate; 3, strong) and the percentage of positively stained cells (0, 0–5%; 1, 6–25%; 2, 26–50%; 3, 51–75%; 4, >76%) was adopted [31]. The staining index (SI) was calculated by multiplying the product of the staining intensity score and the proportion of positive tumor cells.

The cutoff value for high and low expression was determined based on a heterogeneity value measured through log-rank statistical analysis with respect to overall survival [32].

### 2.5. RNAi Knockdown

Cells were transfected with BRF2 siRNA (Qiagen, Valencia, CA) or control siRNA by HiPerFect transfection reagent (Qiagen). The siRNA transfection was performed using Lipofectamine 2000 (Invitrogen) according to the manufacturer's instructions. The following siRNA sequences were used in this study:

 BRF2 siRNA1:
 sense 5-GCACUUACAUGCAGAUAGUTT-3;
 antisense 5-ACUAUCUGCAUGUAAGUGCTT-3;
 BRF2 siRNA2:
 sense 5-GGUGGGAAAUAAUUCCUUATT-3;
 antisense 5-UAAGGAAUUAUUUCCCACCTT-3;
 BRF2 siRNA3:
 sense 5-GCCACCAACAUUUGAGGAUTT-3;
 antisense 5-AUCCUCAAAUGUUGGUGGCTT-3;
 negative control:
 sense 5-UUCUCCGAACGUGUCACGUTT-3;
 antisense 5-ACGUGACACGUUCGGAGAATT-3.

RNA and protein were obtained 48 h after transfection for qRT-PCR or western blot analysis. The BRF2 siRNA that could most effectively deplete BRF2 was used in the following experiments.

### 2.6. Real-Time PCR (RT-PCR)

Surgical specimens were processed immediately after operation. Total RNAs were extracted from tissues by using Trizol reagent (Invitrogen, Carlsbad, USA) according to the manufacturer's protocol and treated with RQ1 RNase-free DNase (Promega); cDNA was synthesized. The expressions of BRF2 and HIF-1*α* were quantified by real-time polymerase chain reaction (PCR) using a Bio-Rad iQ5 real-time PCR system with EvaGreen Supermix (Bio-Rad, Hercules, USA) according to the instruction manual. The primer for GAPDH and BRF2 is as follows: GAPDH, forward, 5′-AGGTCGGTGTGAACGGATTTG-3′, reverse, 5′-TGTAGACCATGTAGTTGAGGTCA-3′; BRF2, forward, 5′-GTGAAGCTCCTGGGACTGGAT-3′, reverse, 5′-GTATTTGGCTGGCACAGAAGG-3′; results are mean ± standard error mean (SEM) of 3 repeat experiments and GAPDH was used as a reference transcript.

### 2.7. Western Blotting Analysis

The fresh tissues were washed three times with ice-cold phosphate-buffered saline (PBS) and lysed on ice in RIPA (radio immunoprecipitation assay) buffer (Cell Signaling Technology, Danvers, MA, USA) containing complete protease inhibitor cocktail (Roche Applied Science, Mannheim, Germany). Protein from tissues or cells was separated via SDS-PAGE and transferred to a PVDF membrane (GE healthcare, USA). Membranes were blocked with 5% fat-free milk in Tris-buffered saline containing 0.1% Tween-20 (TBST) for 1.5 h at room temperature; the membranes were then incubated overnight at 4°C with anti-BRF2 (1 : 1000, Abcam, Cambridge, MA, USA), anti-E-Cadherin (1 : 1000, Cell Signaling, Danvers, MA, USA), anti-N-Cadherin (1 : 1000, Cell Signaling, Danvers, MA, USA), anti-snail (1 : 1000, Cell Signaling, Danvers, MA, USA), or anti-GADPH (1 : 1000, Abcam, Cambridge, MA, USA) antibodies. Followed by anti-rabbit horseradish peroxidase conjugated IgG, an ECL kit (GE healthcare, USA) was used for detection.

### 2.8. Wound Healing Assay

The wound healing assay was performed as described previously [33, 34]. Briefly, cells were subcultured in six-well plates at a density of 1 × 105 cells/well. Upon >80% confluence, the cell monolayer was gently scraped with a yellow pipette tip to generate a linear wound and washed twice with serum-free medium to remove cell debris. Photos were subsequently taken at 0, 12, 24, and 36 h. The closure of the wounds was enumerated by the distance of cells moved into the wounded area. The experiment was repeated twice with triplicate measurements in each experiment.

### 2.9. Transwell Assay

The invasion assay was performed using transwell chambers (Corning, New York, USA) with 50 *μ*L Matrigel-precoated (BD, San Diego, USA) polycarbonate membrane (8.0 *μ*m pore size) as described in the previous report [35]. Briefly, cells were collected and resuspended in the serum-free RPMI 1640 medium at a concentration of 1 × 10^5^ cells/mL, respectively. Then, the cell suspensions were added into the top chambers (200 *μ*L/well) and the bottom chambers were filled with RPMI 1640 medium containing 10% FBS (600 *μ*L/well), followed by a 24 h incubation at 37°C. The cells that did not penetrate the polycarbonate membrane were swabbed using cotton bud; then, the cells that transmembraned through and adhered to the bottom of polycarbonate membrane were stained with 4′,6-diamidino-2-phenylindole dye (1 *μ*g/mL) for 10 min, photographed under an Olympus fluorescence microscope, and counted manually. The average of three randomly selected ×400 fields' cell counts was recorded as the value of each chamber. The experiment was repeated twice with triplicate measurements in each experiment.

### 2.10. Statistical Analysis

Chi-square test was used to test the associations between BRF2 expression and clinicopathological factors. The correlation between BRF2 protein immunoreactivity and E-Cadherin, N-Cadherin, or snail was analyzed by nonparametric test (Mann-Whitney *U* test or Kruskal-Wallis *H* test). Kaplan-Meier method was used to calculate the survival curves, and log-rank test was used to compare the difference between the survivals of patient subgroups. Multivariate Cox regression analysis was used to identify significant independent prognostic factors. The in vitro observations were analyzed using the Student's *t*-test. The data from at least two independent experiments were expressed as the mean ± standard deviation (SD). Differences between groups were considered significant for *P* value <0.05. All statistical analyses were performed with SPSS 17.0 statistical software (SPSS Inc., Chicago, IL, USA).

## 3. Results

### 3.1. BRF2 Expression in NSCLC

**Figure 1:**
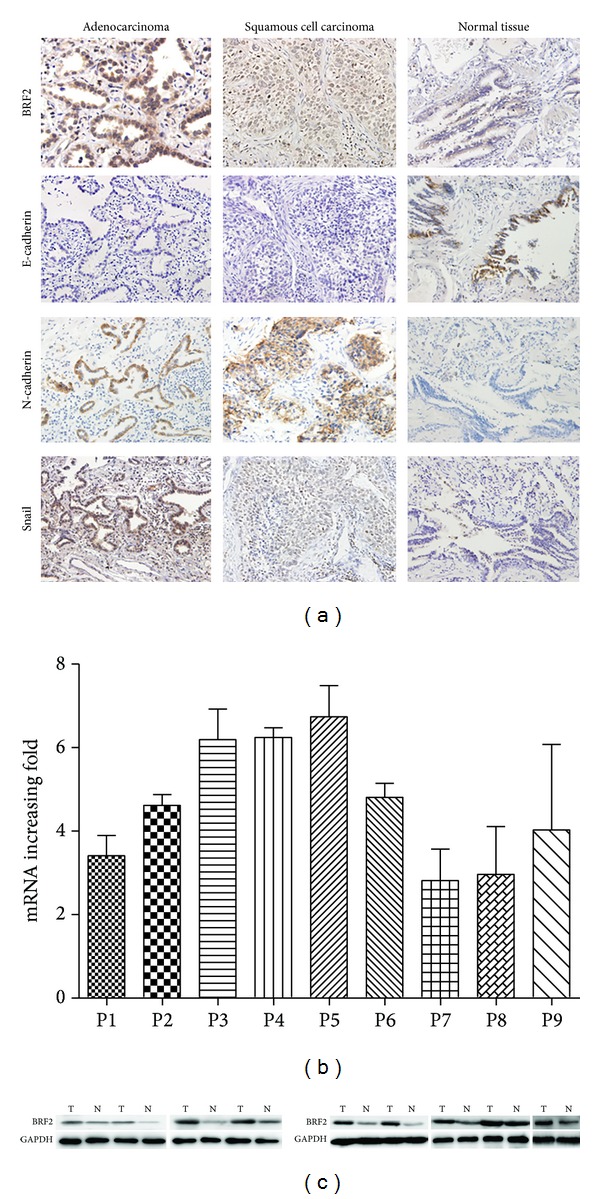
The expression pattern of BRF2 in NSCLC tissues. (a) Immunohistochemical analysis of BRF2, E-cadherin, N-cadherin, and snail in nonsmall cell lung carcinoma (×400). (b) Quantitative real-time PCR analyses of BRF2 mRNA in eight pairs of matched NSCLC and noncancerous tissues with GAPDH as a loading control in both panels. (c) Protein levels of BRF2 expression were evaluated by western blotting from paired noncancerous tissue and NSCLC (T: tumor tissue; N: normal tissue).

We detected the expression of BRF2 protein in the normal lung tissues, adjacent nontumor tissues, and tumor tissues by immunohistochemistry. As shown in Figure 1(a), diffuse nuclear staining of BRF2 protein was observed in cancer cells, but BRF2 was barely detected in normal lung tissues. In addition, some staining was observed in the cytoplasm of cancer cells. However, we observed no statistically significant correlation between BRF2 protein expression and any clinicopathological features of NSCLC tissues (*P* > 0.05, Table 1). The mean value of BRF2 overexpression in 77 NSCLC tissues was 50.65%, significantly higher than that in adjacent tissues and normal lung tissues (32.43% and 30.23%, resp.; *P* = 0.046; Table 2).

To investigate the status of BRF2 gene expression in NSCLC, we used real-time PCR to measure the mRNA expression in 14 pairs of primary cancer tumors and adjacent noncancerous specimens. Compared with their adjacent noncancerous specimens, 9 of 14 NSCLC had upregulated expression (Figure 1(b)). Consistently, western blot analysis showed that the 9 cases also had higher BRF2 protein expression than adjacent tissues (Figure 1(c)).

### 3.2. High BRF2 Expression Predicts Poor Prognosis in NSCLC Patients

**Figure 2:**
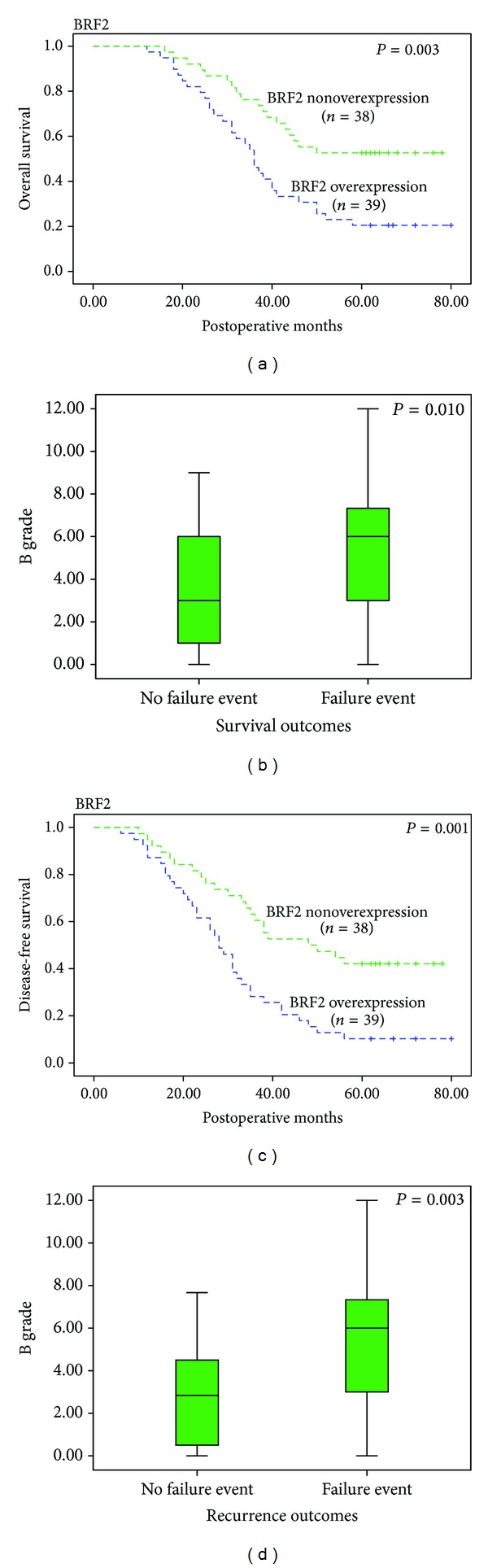
Kaplan-Meier curves of disease-free and overall survival according to the status of BRF2 protein expression (a, c). Mann-Whitney *U* test demonstrated that tumors of failure event patients showed significantly higher BRF2 score grade than tumors of no failure event patients (b, d).

Of the 77 patients, 49 (63.63%) cases died within 5 years after operation, and tumor relapse developed during follow-up in 57 (74.03%) patients. Kaplan-Meier analyses compared by the log-rank test were used to calculate the effect of the clinicopathologic factors with lung cancer on overall survival and disease-free survival. Univariate analysis demonstrated that BRF2 protein overexpression (*P* = 0.001), developing clinical stage (*P* < 0.001), and E-cadherin protein overexpression (*P* = 0.001) significantly predicted decreased overall 5-year survival and a higher risk of recurrence. Furthermore, the multivariate analyses identified BRF2 protein overexpression (*P* = 0.025), clinical stage (*P* = 0.017), E-cadherin protein overexpression (*P* = 0.020), and the differentiation of cancer (*P* = 0.046) as independent prognostic factors for progression-free survival. Meanwhile, multivariate analysis identified BRF2 overexpression as an independent prognostic factor for overall survival (*P* = 0.001; Figures 2(a) and 2(c); Table 3); we analyzed the correlation between BRF2 protein immunoreactivity and the survival outcomes and relapse outcomes by Mann-Whitney *U* test. Consistently, patients in the mortality and recurrent groups demonstrated a high level of BRF2 protein expression during follow-up period (*P* = 0.01 and 0.003, resp.; Figures 2(b) and 2(d)).

**Figure 3:**
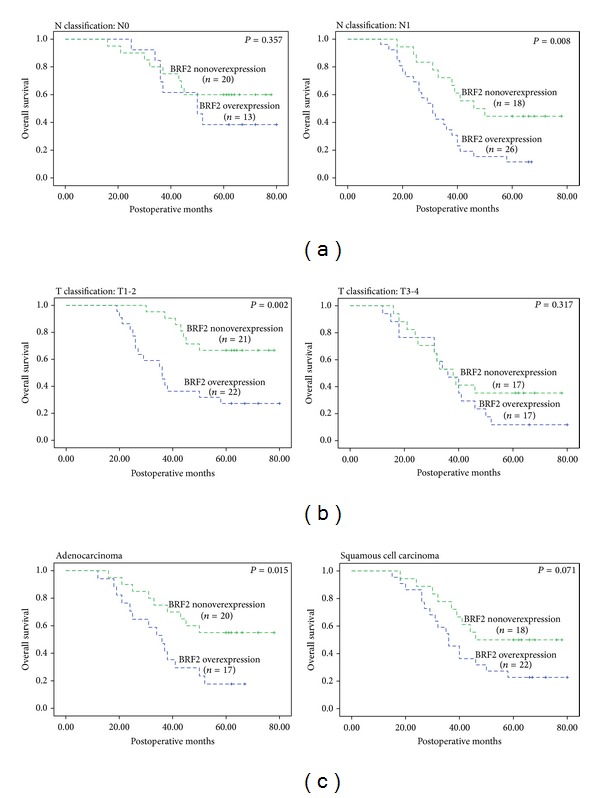
Kaplan-Meier survival curves of patients stratified according to lymph node metastasis (a), tumor size (b), and pathological type (c).

We further analyzed the prognostic significance of BRF2 protein in selective patient subgroups stratified according to the clinicopathologic factors of ESCC. Univariate analysis demonstrated that the overall 5-year survival rate of patients with BRF2 protein high expression was significantly lower than that of the remaining patients among N1, T1-2, and adenocarcinoma (*P* = 0.008, *P* = 0.002, and *P* = 0.015, resp.; Figure 3).

### 3.3. Correlation between Expression of BRF2 and the EMT Marker Proteins

**Figure 4:**
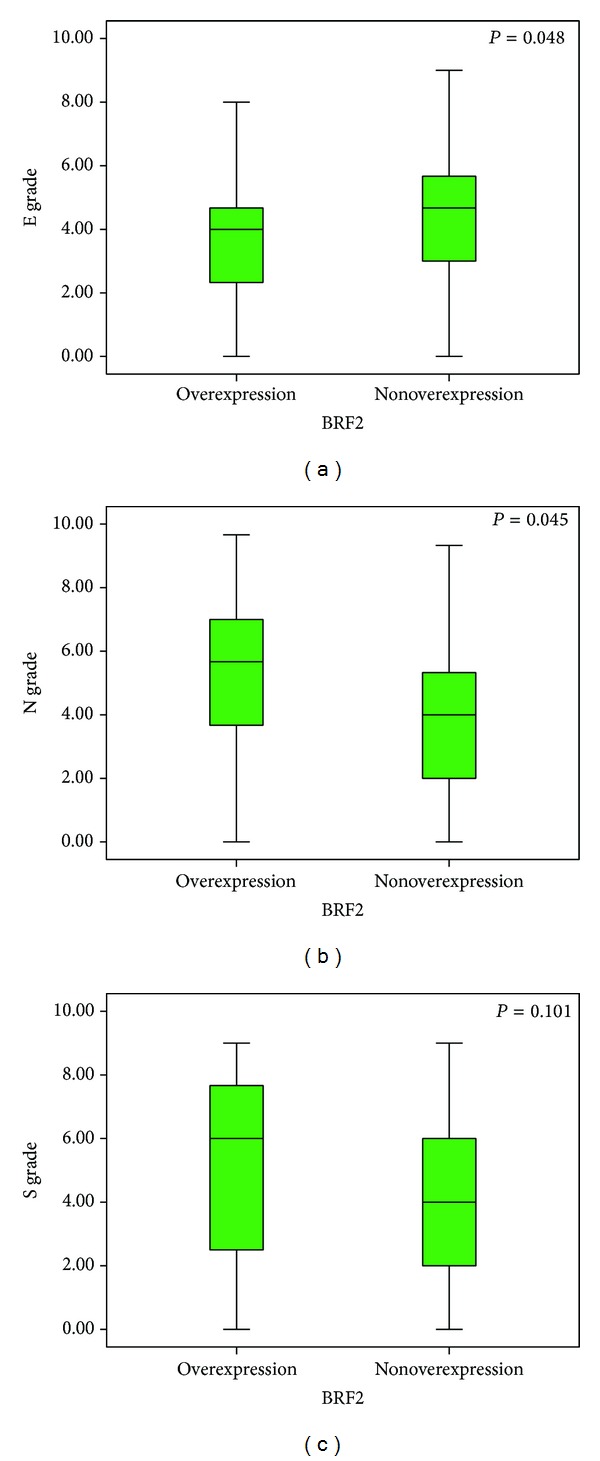
Epithelial-mesenchymal transition factors score grade in relation to BRF2 protein immunoreactivity. Mann-Whitney *U* test demonstrated that tumors with BRF2 protein high expression showed lower E-cadherin score grade and higher N-cadherin and snail score grade than tumors with BRF2 protein low expression (*P* = 0.048, 0.045, and 0.101, resp.).

Typical immunohistochemical staining patterns observed for the EMT marker proteins in NSCLC are shown in Figure 1(a). Positive expression of E-Cadherin and N-Cadherin was localised to the cell membrane, and positive expression of snail was localised to the cell nucleus. The mean values of E-cadherin expression, N-cadherin expression, and snail in 77 NSCLC tissues were 27.27%, 49.35%, and 51.94%, respectively. The correlations between BRF2 protein immunoreactivity and E-Cadherin, N-Cadherin, or snail were analyzed by Mann-Whitney *U* test, and the result also showed that high expression of BRF2 correlated with a loss of E-Cadherin expression (*P* = 0.048) and anomalous positivity of N-Cadherin (*P* = 0.045); we also found such a trend in the correlation between high expression of BRF2 and positivity of snail in clinical NSCLC samples, despite the fact that the statistical significance was not reached. (*P* = 0.101; Figure 4).

### 3.4. Downregulation of BRF2 Protein Decreased the Migration and Invasion of NSCLC Cells

**Figure 5:**
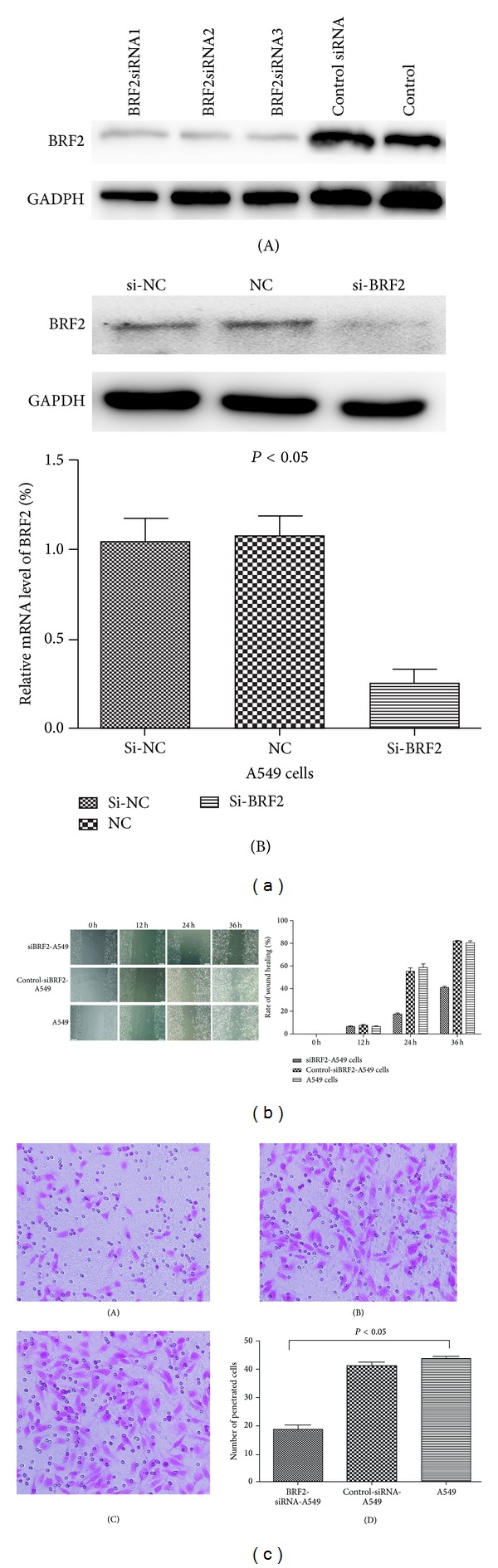
Targeted knockdown of BRF2 expression inhibited the metastatic potentials of NSCLC A549 cells. (a): (A): the levels of BRF2 protein in the A549 cells separately treated with different siRNAs; (B): the detection of the knockdown of BRF2 in A549 cells by WB and RT-PCR, respectively (*P* < 0.05). (b) The representative pictures of wound healing assay (×200) and the quantitative analysis of the migration potential of A549 cells (*P* < 0.05). (c) Downregulation of BRF2 protein decreased the invasion of A549 cells Transwell invasion assay: (A) BRF2-siRNA A549 cells; (B) control-siRNA A549 cells; (C) A549 cells (×400); (D) the quantitative analysis of the invasion potential of A549 cells (*P* < 0.05).

To find out whether BRF2 mediates prognosis in lung cancer through promoting metastasis, we applied small interfering RNA technology to knock down the BRF2 expression on the migratory and invasive ability of A549 and SK-MES-1 cells (Figure 5(a)). The results suggested that the disruption of BRF2 expression could significantly inhibit the migratory and invasive abilities of NSCLC cells by wound healing assay and transwell migration assay (Figures 5(b) and 5(c)).

### 3.5. BRF2 Downregulated Expression Induced Loss of the Epithelial-Mesenchymal Transition of NSCLC Cells

**Figure 6:**
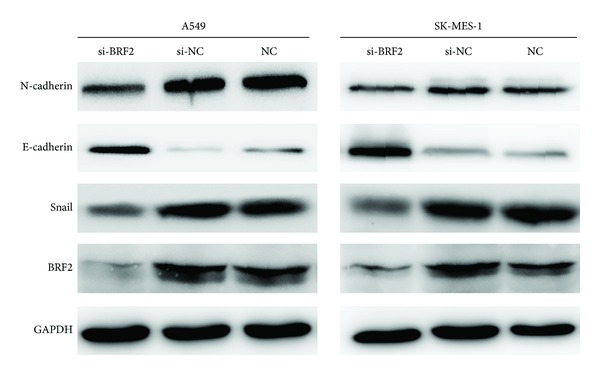
Targeted knockdown of BRF2 expression resulted in a gain of E-cadherin and a loss of N-cadherin and snail in A549 cells and SK-MES-1 cells. The expression of E-cadherin, N-cadherin, and snail was detected by western blot analysis. GADPH was used as loading control.

Enhanced cell migration and invasion properties are important consequences of EMT [36]. Given the fact that BRF2 significantly increased the cell migration and invasion, we hypothesized that BRF2 may play an important role in the EMT process. To investigate whether BRF2 regulates the EMT transition in lung cancer cells, we examined the expression of the key EMT markers E cadherin and N-cadherin in BRF2 knocked down lung cancer cells by western blotting analysis. We found that knockdown of BRF2 led to decreased expression of N-cadherin. Meanwhile, the expression of epithelial markers such as E-cadherin was elicited in response to BRF2 silencing. The expression of E-cadherin is negatively regulated by transcription factors, such as snail. Therefore, we investigated whether BRF2 downregulated expression suppresses EMT through the downregulation of the E-cadherin repressors snail. Interestingly, the protein expression levels of snail were markedly suppressed in BRF2 downregulated expression cells compared to control cells (*P* < 0.05; Figure 6). Collectively, these results imply that BRF2 plays a critical role in the process of EMT.

## 4. Discussion

The ability to proliferate uncontrollably is the dominant characteristic of many types of cancer cells. And it has been demonstrated that overexpression of tRNA^iMet^, which were responsible for RNA polymerase (pol) III, induces proliferation and immortalization of fibroblasts [37]. BRF2 protein is encoded by a gene located on chromosome 8p12, one of the most frequent amplification events in NSCLC. In addition, BRF2-TFIIIB was involved in the process of the RNA pol III transcription deregulation [28]. Several studies have shown that BRF2 is overexpressed in several types of cancer and suggest the oncogenic role of BRF2 [38, 39]. In the present study, we assessed the expression levels of BRF2 using IHC staining, RT-PCR, and western blot in a cohort of NSCLC patients and for first time demonstrated that elevated expression of BRF2 predicted an unfavorable prognosis in NSCLC patients. Moreover, we demonstrated for the first time that BRF2 promotes cell metastatic and invasive capacities by inducing EMT phenotype in lung cancer cells.

In this study, our results showed that there was no significant correlation between BRF2 expression and the clinicopathological features of NSCLC in the statistical analysis. Notably, our survival analysis demonstrated that BRF2 protein overexpression significantly predicted decreased overall 5-year survival and higher recurrence rate. Moreover, further analysis using the Cox regression model confirmed that BRF2 expression was an independent factor in predicting progression-free survival for NSCLC patients, suggesting that BRF2 protein may be potential prognostic factors for the relapse of NSCLC patients. So we conclude that BRF2 possesses a certain effect in invasion and metastasis of lung cancer and is an independent prognostic factor of recurrence and metastasis in lung cancer. Considering the oncogenic role of BRF2 in the tumor cell, Lockwood et al. identified BRF2 as a novel lineage-specific oncogene in lung squamous cell carcinoma. In our study, however, we found that BRF2 plays critical role in prognosis in 37 patients with AC; however, the log-rank test showed a trend towards better overall survival in the high BRF2 group in 40 patients with SCC but this did not reach statistical significance (*P* > 0.05). However, underlying mechanisms need to be further explored.

EMT is a key process in tumor metastasis [40, 41] and is a process, whereby epithelial cells lose their epithelial cell features and acquire a mesenchymal morphological phenotype that is associated with some characteristic alterations at the molecular levels. Loss of cell junctions is considered as a crucial step in the progression of tumor invasion. E-cadherin contributes to the occurrence of EMT by disruption of adherens junction complex [42]. Our data revealed that inhibition of BRF2 could suppress the metastatic ability, while knocking down of BRF2 expression by siRNA inhibited the activity of tumor cell migratory and invasive properties in vitro and is companied by upregulated expression level of E-cadherin and downregulated expression level of N-cadherin. Interestingly, we also found this correlation in lung cancer tissues using immunohistochemistry and western blot.

Snail is one of the transcriptional regulators of this E-cadherin to N-cadherin switch [43]. Moreover, snail binds to consensus E-box sequences in the E-cadherin gene promoter and downregulates E-cadherin transcription. We provide evidence that downregulating BRF2 suppressed EMT associated with the expression transcription factors snail in vitro. However, we only found a trend towards the correlation between upregulated expression level of BRF2 and downregulated expression level of snail in cancer tissues by Mann-Whitney *U* test (*P* > 0.05). We hypothesize that multiple regulation factors overshadowing the snail role in regulating the E-cadherin in lung tissues may be the reason.

In conclusion, this study demonstrates that the overexpression of BRF2 in NSCLC is a strong indicator of more aggressive tumors and poor clinical outcome. Moreover, overexpression of BRF2 could increase the migratory and invasive abilities of NSCLC cells. These results suggest that the BRF2 expression is critical for the invasiveness of malignant NSCLC cells, possibly through EMT involving upregulation of snail and consequent aberrant expression of E-cadherin and N-cadherin. Thus, it may be a candidate biomarker for NSCLC prognosis and a target for new therapies.

## Figures and Tables

**Table 1:** The correlation of clinicopathologic variables of NSCLC with BRF2 expression.

BRF2 (overexpression)				
Variable	No. of patients	No	Yes	*P* ^a^
Age				0.424
≤65 years	38	17	21	
>65 years	39	21	18	
Gender				
Male	46	26	20	0.125
Female	31	12	19	
Smoking				
No or little	60	30	30	0.830
more	17	8	9	
Histology				0.427
Adeno	37	20	17	
Squamous	40	18	22	
Differentiation				0.210
Well	35	14	11	
Moderate	18	11	7	
Poor	34	13	21	
T classification				0.437
T1	17	9	8	
T2	26	12	14	
T3	24	14	10	
T4	10	3	7	
N classification				0.087
N0	33	20	13	
N1	44	18	26	
Clinical stage				0.839
I	20	11	9	
II	32	15	17	
III	25	12	13	

^a^ *P* chi-square test.

**Table 2:** The expression of BRF2 in lung cancer, adjacent lung cancer tissues and normal tissues.

Variable	*n*	Positive (*n*)	Positive rate (%)	*χ* ^2^	*P*
Cancer tissue	77	39	50.65	6.155	**0.046**
Adjacent tissue	37	12	32.43		
Normal tissue	43	13	30.23		

**Table 3:** Univariate and multivariate analyses of prognostic variables.

Variable	*P* value (log-rank test)	95.0% confidence interval	Exp (*B*)	*P* value
	PFS Univariate analysis	PFS Multivariate analysis		
Gende	0.923	0.855–3.389	1.702	0.130
Age	0.353	0.423–1.748	0.860	0.677
Smoking	0.243	0.465–2.472	1.072	0.871
Histology	0.792	0.346–1.229	0.652	0.186
T status	**0.040**	0.283–1.089	0.555	0.087
Clinical stage	**0.000**	2.177–19.997	6.597	**0.001**
Differentiation	0.139	0.521–1.129	0.767	0.178
BRF2 protein	**0.001**	0.121–0.561	0.260	**0.001**
E-cadherin	**0.001**	1.192–5.534	2.568	**0.016**
N-cadherin	0.919	0.905–3.327	1.735	0.097
Snail	0.295	0.548–2.124	1.079	0.826

	OS Univariate analysis	OS Multivariate analysis		
Gender	0.780	0.691–3.018	1.444	0.328
Age	0.296	0.507–2.334	1.088	0.829
Smoking	0.370	0.369–2.146	0.890	0.795
Histology	0.878	0.500–1.902	0.975	0.941
T status	0.082	0.327–1.492	0.698	0.354
Clinical stage	**0.001**	1.267–14.989	4.357	**0.020**
Differentiation	0.057	0.410–0.992	0.638	**0.046**
BRF2 protein	**0.003**	0.168–0.888	0.387	**0.025**
E-cadherin	**0.001**	1.215–7.613	3.042	**0.017**
N-cadherin	0.381	0.618–2.525	1.250	0.535
Snail	0.221	0.575–2.579	0.698	0.354
